# IL-21: A Pleiotropic Cytokine with Potential Applications in Oncology

**DOI:** 10.1155/2015/696578

**Published:** 2015-04-15

**Authors:** Michela Croce, Valentina Rigo, Silvano Ferrini

**Affiliations:** Department of Integrated Oncological Therapies, IRCCS AOU San Martino-IST, Istituto Nazionale per la Ricerca sul Cancro, 16132 Genoa, Italy

## Abstract

Interleukin- (IL-) 21 is a pleiotropic cytokine that regulates the activity of both innate and specific immunity. Indeed, it costimulates T and natural killer (NK) cell proliferation and function and regulates B cell survival and differentiation and the function of dendritic cells. In addition, IL-21 exerts divergent effects on different lymphoid cell leukemia and lymphomas, as it may support cell proliferation or on the contrary induce growth arrest or apoptosis of the neoplastic lymphoid cells. Several preclinical studies showed that IL-21 has antitumor activity in different tumor models, through mechanism involving the activation of NK and T or B cell responses. Moreover, IL-21's antitumor activity can be potentiated by its combination with other immune-enhancing molecules, monoclonal antibodies recognizing tumor antigens, chemotherapy, or molecular targeted agents. Clinical phase I-II studies of IL-21 in cancer patients showed immune stimulatory properties, acceptable toxicity profile, and antitumor effects in a fraction of patients. In view of its tolerability, IL-21 is also suitable for combinational therapeutic regimens with other agents. This review will summarize the biological functions of IL-21, and address its role in lymphoid malignancies and preclinical and clinical studies of cancer immunotherapy.

## 1. Introduction

**Figure 1:**
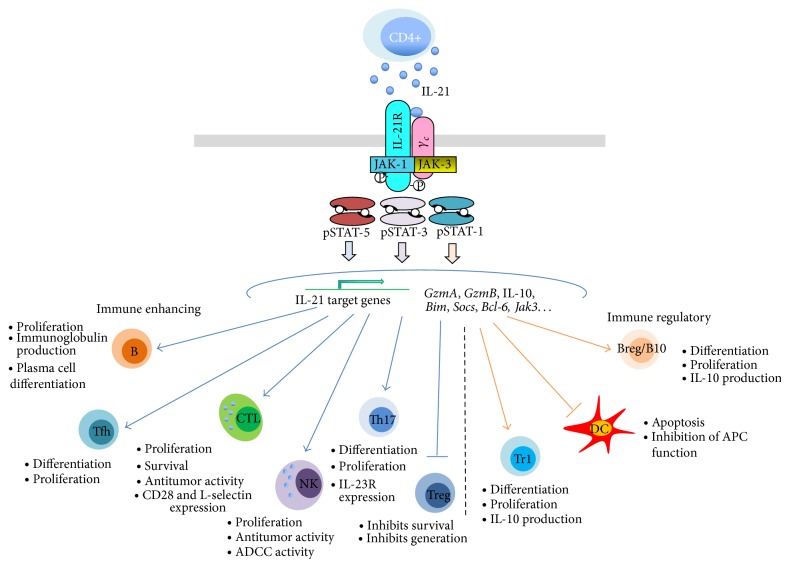
Schematic representation of IL-21 signaling pathways and its main biological effects on different target cells.

Interleukin- (IL-) 21 was identified in 2000 as a CD4^+^ T cell-derived cytokine and the ligand of an IL-2R*β*-related orphan receptor [1, 2], now defined as IL-21R. IL-21 is a member of the cytokine family sharing the common gamma chain in their receptor complexes [3, 4] that comprises IL-2, IL-4, IL-7, IL-9, and IL-15. These cytokines display a similar four-helix bundle structure and functional redundancy in the regulation and homeostasis of the lymphoid system, although each member also has specific functions. IL-21 binding to its high-affinity receptor complex, formed by the IL-21R chain and the *γ*c, triggers different signal transduction pathways, which leads to the transcriptional regulation of a distinct set of genes [5]. An early event triggered by IL-21 engagement is the activation of the Janus kinase- (JAK-) 1 and JAK-3 that phosphorylate tyrosine residues the intracellular regions of the receptor chains [6] (Figure 1). These phosphorylated regions serve as docking sites for the SH2 domains of specific signal transducers and activators of transcription (STAT) proteins, including STAT-1, STAT-3, and, to a lesser extent, STAT-5 [7, 8]. Once recruited, STATs are phosphorylated by JAK kinases and then detach from their docking sites, dimerize, and migrate into the nucleus to activate specific gene promoters. Besides the JAK/STAT pathway, IL-21 also activates the phosphoinositide 3-kinase/protein kinase B (PI3K/AKT) and the ras/raf/Mitogen-activated protein kinase kinase (MEK)/mitogen-activated protein kinases (MAPK) pathways [7]. In addition, IL-21 signaling involves interferon-regulatory factor 4 (IRF4), which cooperates with STAT3 for transcriptional activation of several target genes [9]. Among IL-21 sensitive genes,* Gzma* and* Gzmb* encode for granzymes, involved in the activity of cytotoxic T lymphocytes (CTL) and natural killer (NK) cells. Other IL-21-activated genes such as* Bcl6*,* Bim* [10], and* Prdm1* (encoding for Blimp1) [11] are major regulators of B lymphocyte survival and differentiation, while suppressor of cytokine signaling- (*SOCS*-) * 1* and* SOCS-3* encode for negative-feedback regulators of cytokine receptor signaling [12].

## 2. Costimulatory Activities of IL-21 on B, T, and NK Cells

Early studies showed that IL-21 costimulates the proliferation of B, T, and NK cells and mediates the differentiation of activated NK cells into more potent effector cells [2] (Figure 1), suggesting that IL-21 may represent a potentially useful agent for the development of tumor immunotherapies.

### 2.1. Effects on B Cells

IL-21 exerts complex effects on human and mouse B cell proliferation and survival as it can mediate apoptosis of B cells activated via toll like receptor (TLR) signals [10, 13]. On the contrary it induces B cell proliferation in the presence of appropriate cosignals delivered by B cell receptor (BCR) stimulation and CD40 ligand (L) expressed by T helper (Th) cells [10, 14]. Moreover, IL-21 induces the differentiation of B lymphocytes into plasma cells through the induction of Blimp1 expression in vitro and in vivo [9, 11]. IL-21 also upregulated IgG1 and IgG3 production in vitro by CD40-activated human B cells, and this effect is enhanced by IL-4 costimulation [15]. Several studies suggested that IL-21-dependent signaling via STAT3 is required for the generation of long-lived plasma cells and for T cell-dependent antigen responses and memory (reviewed in [16]). The study of* Il21r*-deficient mice showed that IL-21 plays a nonredundant role in regulating B cell responses, as these mice have reduced IgG1 and enhanced IgE responses upon antigen challenge [17]. A recent study reported that homozygous loss-of-function mutations in the* IL21R* gene cause a primary immunodeficiency in humans, resulting in recurrent respiratory and gastrointestinal cryptosporidium infections, and chronic liver disease. Patients showed increased serum IgE levels, in some cases associated with reduced IgG [18]. Similar B cell defects associated with decreased serum IgG and increased IgE levels have been reported in a patient with* IL21* deficiency, who presented with early-onset inflammatory bowel disease and later pulmonary infections [19]. Altogether these data indicate an essential role of IL-21 in regulating B cell responses, although also variable T cell and NK cell defects were reported in* IL21R*-deficient patients.

Several observations indicate an important role of IL-21-driven B cell responses in autoimmune syndromes such as systemic lupus erythematosus (SLE). Indeed, elevated IL-21 expression drives pathogenic autoantibody production in BXSB-Yaa [20] and in the MRL-Fas(lpr) mouse lupus models [21]. IL-21 is also elevated in the skin and plasma of SLE patients, and polymorphisms in the IL-21 gene associate with SLE development [22].

Finally, the B cell enhancing properties of IL-21 may be also important for the induction of antitumor immunity. For example, IL-21 gene transfer promoted the production of tumor-specific IgG, which may contribute to the therapeutic effect in a syngeneic head and neck squamous carcinoma model [23].

### 2.2. Effects on T Cells

IL-21 stimulates the differentiation of T follicular helper (T_FH_) cells, through the induction of the transcription factors Bcl6 and MAF, which regulate their functional program and their ability to stimulate B cell responses [24, 25]. In addition also the induction of IRF4 plays a crucial role in the IL-21-triggered T_FH_ cell differentiation [26]. Upon antigen presentation, T_FH_ cells produce high levels of IL-21, which induces B cell differentiation in the germinal centers. A recent report indicates that circulating T_FH_ cells have memory-like features and can be reactivated by dendritic cells (DCs) to produce high levels of cytokines, including IL-21. Therefore, circulating T_FH_ may provide rapid and strong helper signals to B cells in response to secondary antigen stimulation [27]. Besides classical T_FH_ cells [28], also a subset of CD4^+^CXCR4^+^ICOS^+^CD162^low^CD40L^+^ T cells mediate IL-21-driven B cell differentiation and pathogenic IgG production in extra-follicular sites of autoimmune mouse strains [29].

IL-21 also contributes to the expansion of Th17 cells acting in concert with other cytokines. Th17 cells are a subset of proinflammatory cells, induced by transforming growth factor (TGF) *β* and IL-6, which produce IL-17 and also IL-21 [30, 31]. Although IL-21 is not necessary for Th17 cell induction, it stimulates Th17 cell expansion by inducing the expression of IL-23R and their responsiveness to this cytokine [31, 32]. The role of IL-21 in Th17 responses may explain its involvement in several inflammatory disorders, as extensively discussed in recent reviews [5, 33]. For example, Th17 and Th1 cells produce IL-21 in the gut of patients with Crohn's disease and ulcerative colitis [34, 35]. Similarly, IL-21 expression is high in the gut of mice with dextran sulphate- (DSS-) induced colitis, whereas* Il21* ^−/−^ mice showed reduced DSS-colitis, lower inflammatory cell infiltration, and IL-17 production [36].

The effects of IL-21 on CTLs are well documented and important for its applications in tumor immunotherapy. For example, IL-21 in combination with peptide-pulsed DC, increases the number of human Melan-A/MART-1-specific CD8^+^ T cells displaying high-affinity and a CD45RO^+^CD28^high^ phenotype upon in vitro stimulation [37]. These data suggested a role for IL-21 in the ex vivo generation of antitumor CTLs for adoptive cell therapies. In addition, IL-21 used in combination with IL-15 maintains CD28 expression on CTLs and their responsiveness to signals through this costimulatory pathway [38]. It also costimulates IL-15-mediated CTL-mediated proliferation and function in vitro and the expansion of both memory and naïve CD8^+^ T cells [39]. IL-21 also regulates CTL cytotoxic activity and several preclinical studies of cancer immunotherapy showed that IL-21 sustains antitumor CTL functions [40].* Il21r* ^−/−^ mice have normal numbers of CD8^+^ T cells; however, they showed reduced CTL responses to a vaccinia-virus encoded antigen, further suggesting a role of IL-21 in CTL responses [39]. A recent study showed that IL-21 mediates the expression of the transcription factor T-bet in CD8^+^ T cells, through STAT1 signaling. Moreover T-bet is necessary for the expression of perforin and granzyme B in response to IL-21, as T-bet defective CTLs are unresponsive to IL-21-induced cytotoxicity [41]. IL-21 induces SOCS1 mRNA expression in CD8^+^ T cells, suggesting that SOCS1 downregulates further responses through the IL-21R. Indeed CD8^+^ T cells accumulate in* Socs1* ^−/−^ mice [42]. In vitro studies also showed that IL-21 inhibited the antigen-induced expression of eomesodermin and IL-2R*α* chain in naive CD8^+^ T cells and their maturation into CD44^+^ effector cells. However, IL-21-stimulated CD8^+^ T cells display long-lasting and potent antitumor effects in mice [43]. IL-21 may also modulate CTL trafficking, though the reexpression of C-C chemokine receptor (CCR)7 on* Citomegalovirus* (CMV) antigen-restimulated CD8^+^ T cells [44].

Due to its CTL-enhancing activities, IL-21 plays an important role in the immune response to pathogens. For example, in lymphocytic choriomeningitis virus (LCMV) infection in mice IL-21 levels increase in the acute phase and persist, at lower levels, in chronic infection. Studies in* Il21r* ^−/−^ [45, 46] or* Il21* ^−/−^ [47] mice indicated that IL-21 is necessary for the long-term maintenance and function of CD8^+^ T cells, which control chronic infection. IL-21 plays a role also in human viral infections, for example, HIV-infection. CD4^+^ T cells are the main source of IL-21 and production of IL-21 is compromised during the course of HIV infection [48]. Interestingly, elite controllers, which can suppress viral replication for long periods without therapy, maintain IL-21 production [49]. IL-21 supports HIV-specific CD8^+^ T cell functions, which control infection [50]. Indeed, IL-21 upregulates perforin and granzyme expression in memory and effector CD8^+^ T cells but did not increase their proliferation and activation [51]. Recent studies showed that, in elite controllers, a peculiar subset of HIV-specific CD8^+^ T cells, which produce IL-21, is enriched and may develop as a consequence of chronic activation of the immune system in HIV patients [52, 53].

### 2.3. Effects on NK Cells

Several reports showed that IL-21 also regulates NK cell functions. Although soluble IL-21 alone does not support NK cell proliferation, it costimulates IL-2 or IL-15-triggered mouse NK cell expansion [54]. In addition, IL-21 induces the differentiation of cytokine-activated murine NK cells to highly efficient effector cells, which express high levels of IFN-*γ* and perforin and are endowed with cytolytic activity [55]. However, IL-21 increases NK cell apoptosis and may limit the persistence of NK responses, suggesting a role of IL-21 in the shift from early innate to adaptive immunity [56]. IL-21 also upregulates natural killer group 2, member D (NKG2D) receptor expression in human, and mouse NK cells through the induction of STAT3 tyrosine phosphorylation [57]. In fact, patients with STAT3 mutations display functional NK cell defects and low NKG2D expression [57]. IL-21 cooperates with IL-7, IL-15, and stem cell factor (SCF) to mediate human NK cell differentiation from CD34^+^ progenitor cells present in the cord blood, through the induction of KIRs, CD2 and of cytolytic functions [58]. Although* Il21r* ^−/−^ mice show no NK cell deficiency and IL-21 is not strictly necessary for the NK cell development from progenitors [59], NK cells from* IL21R*-deficient patients showed variable reductions in their cytotoxic activity against NK-sensitive targets, but normal antibody-dependent cell-mediated cytotoxicity (ADCC) activity [18, 19]. However, IL-21 is able to potentiate ADCC mediated by NK cells in vitro and in vivo, and this effect may be relevant for antibody-based cancer immunotherapy [60, 61]. Importantly, NK cell dysfunction in some cancer patients may impair their ADCC activity, which can be restored by in vitro stimulation with IL-21 [60].

## 3. Dual Role of IL-21 in Immune-Regulation

IL-21 stimulates both innate and adaptive immunity, but on the other hand, it can also mediate negative regulatory effects on lymphoid and myeloid cells. In general the effects of IL-21 on immune regulation are multifaceted as it activates some immune-regulatory pathways whereas it inhibits others (Figure 1). These effects have important implications both in autoimmunity and in cancer.

### 3.1. Effect on Dendritic Cells (DC)

IL-21 inhibits DC activation and maturation. Indeed, in the presence of granulocyte macrophage colony-stimulating factor (GM-CSF), IL-21 leads to the differentiation of altered DCs with reduced capacity to prime T cell activation in vitro [62]. IL-21 can also induce apoptosis of conventional DCs via STAT3 and Bim, but this effect may be partially inhibited by GM-CSF [63]. Therefore, IL-21 hits the early phase of the immune response, by blocking antigen-presenting cells (APC).

### 3.2. Induction of IL-10 Expression in T Cells

IL-21 may have immunosuppressive functions also through a STAT3-dependent induction of IL-10 expression [64]. IL-10 is an immunosuppressive cytokine known to subdue inflammatory responses and inhibit the functions of APC and T cell response to antigens [65]. Indeed, T cells from* Il21* transgenic mice produced high levels of IL-10 upon stimulation with anti-CD3 + anti-CD28 monoclonal antibodies (mAbs), while T cells from* Il21r* knockout (KO) mice produce less IL-10, relative to wild type (wt) mice. Moreover, wt Th1 cells, primed in the presence of IL-21, produced IL-10 and suppressed proliferation of OT-1 transgenic mouse CD8^+^ T cells, stimulated in the presence of the specific ovalbumin peptide in vitro [64]. Another report showed that IL-6 induced the generation of mouse IL-10-secreting type 1 regulatory T (Tr1) cells, through the stimulation of IL-21 production by CD4^+^ T cells in vitro. In this model, IL-21 cooperates with IL-2 to induce IL-10 production in CD4^+^ T cells [66]. Moreover, in human cord blood, IL-21 polarizes naïve CD4^+^ T cells into IL-10-producing cells, thus affecting the balance between effector and regulatory functions during inflammation in human newborn [67]. IL-27 is also an inducer of Tr1 cells in the mouse. This effect is mediated by IL-21 production, in concert with induction of the transcription factor c-MAF and the costimulatory receptor ICOS [68]. IL-21 may then act as an autocrine growth factor for Tr1 cells.

### 3.3. Induction of B-10 or B-Regulatory (Breg) Cells

Different types of Breg cells have been described [69]. Among these, B-10 cells are IL-10-producing B cells, induced by IL-21 stimulation. In particular, in a mouse model of multiple sclerosis, IL-21 and CD40 interaction with T cells induce B cell differentiation into regulatory, CD1d^high^CD5^+^ B-10 cells, which express IL-21R and secrete large amounts of IL-10. These in vitro generated B-10 cells, if transferred in vivo in mice with experimental autoimmune encephalomyelitis (EAE), suppress the autoimmune response and inhibit disease symptoms [70]. Another report showed that IL-21, in concert with TLR or BCR stimulation, induced B cells to acquire a CD19^+^CD38^+^CD1d^+^IgM^+^CD147^+^ granzyme B^+^ regulatory phenotype, together with the expression of other immune-regulatory molecules such as IL-10, CD25, and indoleamine-2,3-dioxygenase. B cells with a similar regulatory Breg phenotype are present in human tumor infiltrates and, therefore, may contribute to local suppression of antitumor immune responses [71].

### 3.4. Inhibition of Regulatory T (Treg) Cells

Opposite to these supportive effects on IL-10 production and induction of Breg cells, IL-21 does not support but rather inhibits regulatory T (Treg) cells. Indeed IL-21, different from IL-2, is not able to sustain Forkhead box P3 (FOXP3)^+^CD4^+^CD25^high^ mouse Treg proliferation and activity in vitro [72]. Moreover, IL-21 supports the proliferation of human CD4^+^CD25^−^ T cells and neutralizes the suppressive effects of Treg cells without altering their survival. The authors concluded that IL-21 induces resistance in CD4^+^CD25^−^ T cells to Treg-mediated suppression and suggested a mechanism by which IL-21 could support T cell responses in immune-mediated diseases [73]. In vivo, IL-21 indirectly inhibits the generation of mouse Treg cells through the inhibition of IL-2 production, which is required for Treg expansion and fitness [74]. Even if IL-21 and IL-2 display different effects on Treg cell development, these two cytokines synergize to sustain T cell proliferation and responses. This cooperative effect of IL-21 is related both to stimulation of T cell proliferation and to inhibition of IL-2/TGF-*β*-driven Treg cell development. In fact, IL-21 induced STAT3 activation, a negative regulator of Treg cell differentiation, and downmodulated Smad2/3, Treg cell inducers [75]. Moreover, in patients with giant cell arteritis, IL-21 is detrimental for Foxp3-expressing Treg cell development and favors Treg cell apoptosis and disease development [76].

The dual role of IL-21 in immune regulation raised a note of caution in the use of therapeutic agents [77] blocking the IL-21/IL-21R axis in autoimmune diseases, as also suggested by preclinical studies in murine lupus models [78]. On the other hand, the activation of immune-regulatory pathways by IL-21 may limit the efficacy of IL-21-based cancer immunotherapy and support the concept that IL-21 may act as a double-edged sword in cancer [5].

## 4. Role of IL-21 in Lymphoproliferative Disorders 

Several lymphoid cell malignancies express IL-21R, but IL-21 exerts divergent effects on cell survival and proliferation, in relationship to the different type of malignancy, stage, and presence of costimulatory signals [79].

### 4.1. Chronic B-Type Lymphocytic Leukemia (B-CLL)

B-CLL cell survival and clonal expansion require a cross-talk with supportive cells within the lymphoid tissue and bone marrow microenvironment [80, 81], which favors CLL progression and drug resistance. Several contact-dependent signals, as well as chemokines and cytokines, play a role in this cross-talk. IL-21R is expressed at variable levels in B-CLL cells and is inversely related to CD38, a marker whose high expression is associated with a poor prognosis [82]. In addition, IL-21R expression is upregulated by stimuli such as CD40L [82] and CpG oligodeoxynucleotide [13], which enhance the IL-21 sensitivity in B-CLL cells. Different from other cytokines of the IL-2 family (e.g., IL-4 and IL-15), which support B-CLL cell proliferation, IL-21 induced apoptosis in vitro through JAK/STAT signaling. In B-CLL cells, IL-21 does not activate the extracellular signal-regulated kinases (ERK) 1/2 pathway, which, instead, is activated by IL-15 and mediated cell survival [8]. IL-21-mediated apoptosis involves caspase-8 and caspase-3 activation and cleavage of their substrates Bid, poly (ADP-ribose) polymerase (PARP) and of p27Kip-1 [82]. In addition, IL-21 also induces the upregulation of the BH3 domain protein Bcl-2 interacting mediator of cell death (BIM), which is also involved in apoptosis of B-CLL cells, as demonstrated by BIM silencing [83]. Another report showed that the combination of IL-21 with either CpG oligodeoxynucleotides or anti-B cell-receptor antibodies induce functional granzyme B expression, in B-CLL cells. Therefore, B-CLL cells not only undergo apoptosis but also induce the killing of untreated bystander cells [13]. Very recent data indicate that also molecules, involved in granzyme B trafficking and processing, are induced by IL-21 + CpG stimulation, whereas the granzyme inhibitor Serpin B9 is downmodulated [84]. Under these conditions, B-CLL cells also display enhanced immunogenicity, cytotoxicity, and features similar to that of killer dendritic cells. The cotreatment of B-CLL cells with IL-21 and fludarabine or the anti-CD20 mAb rituximab augmented the cytotoxic effect of these agents. Moreover, IL-21 increases the ability of NK cells to mediate ADCC against rituximab-coated B-CLL cells in vitro [83]. Altogether these data suggested the possible use of IL-21 in B-CLL therapy, particularly in combination with fludarabine and rituximab. These data were achieved by stimulating CLL cells with pharmacological concentrations (10–100 ng/mL) of IL-21 in vitro and differ from recent findings obtained under more physiological conditions. Indeed, B-CLL cells cocultured with activated T cells proliferate and IL-21 released by activated T cells costimulates B-CLL proliferation, in cooperation with contact-dependent signals and with other cytokines. IL-21 RNA and protein are expressed in CD4^+^CXCR5^+^  T_FH_ cells in lymph nodes, suggesting a supportive role of IL-21 in the B-CLL microenvironment [85]. In addition, IL-21 and IL-4 cooperated to promote B-CLL cell proliferation, and IL-21 increased the size of the “side population,” which is associated with resistance to chemotherapy [86]. These recent data suggest that IL-21 activity in B-CLL differs depending on experimental conditions and raise concerns about the therapeutic use of IL-21 in B-CLL.

### 4.2. Follicular Lymphoma (FL)

IL-21 has been reported to induce apoptosis also in some FL cell lines [87] and in primary FL cells, which constitutively express high levels of IL-21R [88]. IL-21 induced apoptosis in B lymphoma cell lines was related to caspase-8 and -3 activation, Bcl-2 decrease, and proapoptotic Bax increase [87]. However, IL-21 triggered apoptosis only in a minority of cell lines bearing the t(14; 18) translocation, typical of FL. Resistance was due to either reduced IL-21R expression or defective* JAK3* gene expression, in one cell line. Altogether, these data suggested heterogeneity in IL-21 responsiveness in FL [88]. However, a more recent report indicated that reduced expression of IL-21R is associated with increased overall survival in FL and that IL-21R expression is higher in cases of progression of FL to DLBCL. The latter data were suggestive of a possible role of IL-21 in supporting FL progression in vivo [89].

### 4.3. Diffuse Large B Cell Lymphoma (DLBCL) and Mantle Cell Lymphoma (MCL)

CD10^+^ DLBCL cell lines and primary tumors also express the IL-21R and, in these cells, IL-21 induced cell cycle arrest and caspase-mediated apoptosis [90]. Moreover, IL-21 treatment increases survival of mice bearing human DLBCL xenografts. In DLBCL cells, IL-21 induces upregulation of c-Myc expression, through a STAT3-dependent mechanism. C-Myc, in turn, downregulates antiapoptotic Bcl-2 and Bcl-XL protein expression, thus inducing cell death.

MCL is an aggressive B cell tumor, whose cells also display high levels of IL-21R and are sensitive to IL-21-mediated apoptosis. Gene-silencing experiments revealed that STAT1 mediates IL-21-induced apoptosis in MCL cells. IL-21 up-regulated the expression of proapoptotic proteins BCL2-interacting killer (BIK), NIP3, and HARAKIRI, while it downregulated the antiapoptotic proteins BCL-2 and BCL-XL/S [91]. In addition, IL-21 inhibited signaling through nuclear factor-kappaB (NF-*κ*B), which mediates survival signal in MCL cells.

### 4.4. Other Lymphoproliferative Disorders

IL-21 may act as a paracrine growth factor in other lymphoproliferative disorders. In fact, IL-21 supports neoplastic cell proliferation and/or survival in anaplastic large cell lymphoma [92], Hodgkin's lymphoma [93], multiple myeloma [94, 95], Waldenstrom macroglobulinemia [96], adult T cell leukemia/lymphoma [97], cutaneous T cell lymphoma [98], and Sézary syndrome [99]. Obviously, in these tumors the use of IL-21 as immune-enhancing agent should be regarded with caution. Rather, agents targeting the IL-21/IL-21R system or its signaling pathways may be potentially useful for clinical testing.

## 5. Preclinical Studies of IL-21 Cancer Immunotherapy

In view of its immune stimulatory properties on both innate and adaptive immunity, recombinant (r) IL-21 or IL-21 gene transfer has been widely exploited in preclinical models of cancer immunotherapy either alone or in combination with cellular or molecularly defined vaccines, antibodies, other cytokines, or immune checkpoint blockers. These studies demonstrated that IL-21 triggers distinct mechanisms of antitumor immunity, leading to significant therapeutic effect and disclosed synergistic interactions with different molecules.

### 5.1. Mouse Models of IL-21 Immunotherapy

In early studies, IL-21 gene transfer has been used to generate cytokine-secreting tumor cells, in the attempt to trigger antitumor immune responses through a paracrine effect of IL-21. In general, different IL-21-engineered tumor cells lose their tumorigenic potential in syngeneic mice through the induction of CTL and/or NK cell activation or B cell responses. In particular, mouse mammary adenocarcinoma cells releasing IL-21 showed reduced tumorigenicity in syngeneic mice and primed a protective immune response mediated by CD8^+^ CTLs. In addition, interferon- (IFN-) *γ* and third-order CXCL-9, -10, and -11 antiangiogenic chemokines were soluble mediators of the IL-21-driven tumor rejection and mediated antiangiogenic effects [100]. Similar rejection responses, involving CTL and/or NK cells, were observed for IL-21-secreting melanoma, fibrosarcoma [101], colon [102], renal [103], and bladder cancer cells [104]. In some studies, IL-21-producing cells were used as a whole cell vaccine to treat mice previously challenged with wild type tumor cells. These studies showed significant antitumor effects resulting in increased mice survival or even in the cure of a fraction of tumor-bearing mice, through the induction of antigen-specific CTL responses [72, 105]. Differently, in a murine model of glioblastoma, the use of a tumor cell vaccine producing IL-21, injected intracranially, prevented the subsequent growth of glioma tumors and cured most mice bearing early glioma implants. Cured mice were resistant to a secondary tumor challenge since they developed strong antibody responses to glioma cells [105]. A murine IL-21-secreting whole myeloma cell vaccine (mIL-21-Sp2/0) has also been used to prime immune responses against myeloma in vivo. Lymphocytes from mIL-21-Sp2/0-immunized mice infused in mice lymphodepleted by cyclophosphamide treatment induced protective immunity against murine myeloma [106]. Besides tumor cells, also other cell types transduced with IL-21 gene proved to be effective at inducing antitumor responses. In a syngeneic model of melanoma, IL-21 was used in combination with human (h)gp100, a melanocytic lineage differentiation antigen, to transduce DCs. DC-hgp100/mIL21 used as vaccine reduced B16 melanoma growth when injected intratumorally and reduced lung metastases development, through T and NK cell dependent mechanisms [107]. Intravenous injection of human umbilical cord blood stem cells (UCBSC) engineered to express murine IL-21 inhibited the growth of human ovarian cancer cells xenotransplanted in nude mice and prolonged survival. IL-21 released by UCBSC increased CD56^+^ NK cell numbers, cytotoxic activity, and cytokine release in vivo [108].

rIL-21 has also been tested in preclinical applications, and an early study showed that intraperitoneal IL-21 was more effective than IL-2 or IL-15 in a syngeneic thymoma model [109]. In addition, rIL-21 treatment showed efficacy in different tumor models. In two syngeneic models of melanoma and renal cell carcinoma, rIL-21 given intratumorally strongly inhibited tumor growth and increased the frequency of tumor-infiltrating CD8^+^ T cells and mice survival [110]. Interestingly, rIL-21 showed low toxicity and no induction of vascular-leak syndrome, a dose-limiting toxicity of IL-2 [111].

### 5.2. Preclinical Studies of IL-21 Combination Therapies

IL-21 antitumor activities have been exploited in association with several other molecules, which showed additive or synergistic effects [112]. For example, CD4^+^CD25^high^FoxP3^+^ Treg are frequently increased in tumor bearing hosts and contribute to tumor-related immune suppression, since they can be induced by tumor-deriving factors [113, 114]. Therefore, the use of antibodies targeting Treg cells has been exploited in preclinical studies, in combination with IL-21-based immunotherapy. The use of an anti-CD25 mAb in association with an IL-21-secreting mammary carcinoma cell vaccine cured 70% of syngeneic mice from lung micrometastases through the induction of antigen-specific CD8^+^ T cell responses and production of IFN-*γ* [72]. In another syngeneic model, the combination of an anti-CD4 mAb with an IL-21-secerting neuroblastoma (NB) cell vaccine [115] or rIL-21 [116] induced potent CD8^+^ T cell responses and cooperatively cured most mice bearing systemic NB. The anti-CD4 mAb induced a transient depletion of all CD4^+^ T cells, including Treg cells and their precursors. In the absence of CD4^+^ T cell help, CD8^+^ T cell responses could be supported by exogenous IL-21 supply. This IL-21 + anti-CD4 mAb immunotherapy also induced a powerful long-term memory to NB antigens that required not only CD8^+^ T cells but also re-populating/reprogrammed CD4^+^ helper T cells [116].

In addition to Treg cell targeting, another approach to increase the antitumor immune response is based on the use of immune checkpoint blockers. Immune checkpoints consist of inhibitory pathways of the immune system fundamental for self-tolerance and agents blocking such pathways (e.g., anti-cytotoxic T lymphocyte antigen (CTLA)4, anti-programmed death- (PD-) 1, or anti-PD-L1 mAbs) are gaining increasing importance in cancer immunotherapy [117]. In a murine model of hepatocellular carcinoma, IL-21 showed synergistic activity with soluble PD-1. PD-1 is expressed on activated T cells and the binding with its ligand PD-L1, frequently expressed on solid tumors, inhibits proliferation of CTLs and cytokine secretion thus favoring tumor escape and tolerance. Local gene transfer of plasmids inducing the expression of IL-21 and soluble PD-1 significantly inhibited the development of H22 hepatocellular carcinomas in syngeneic mice, through the activation of CTL and NK cell responses, production of IL-2 and IFN-*γ* [118]. In addition, the combination of anti-PD-L1 with rIL-21 showed cooperative antitumor activity, resulting in frequent complete regression, in different mouse tumor models [119].

IL-21 was also effectively combined with a triple agonistic monoclonal antibody cocktail (TrimAb), containing anti-DR5/anti-CD40/anti-CD137 mAbs. Anti-DR5 induces apoptosis of TNF-related apoptosis-inducing ligand- (TRAIL-) sensitive tumor cells, whereas anti-CD40 activates DCs and anti-CD137 costimulates T cells, to induce tumor-specific immunity. The sequential administration of TrimAb and rIL-21 further potentiates the antitumor activity of the TrimAb leading to the induction of strong antitumor T cell responses and cured very advanced tumors [120].

Other reports demonstrated that IL-21 enhances the activity of therapeutic mAbs, which target tumor-associated antigens. Roda et al. [121] showed that IL-21 significantly augmented the NK cell ADCC activity and IFN-*γ* production to trastuzumab-coated cancer cells in vitro. In addition, IL-21 amplified the effects of an anti-HER2/neu mAb against a Her-2-expressing murine tumor in vivo, through an IFN-*γ*-dependent effect. In cynomolgus monkeys human IL-21 potentiated the B cell depletion induced by rituximab (anti-CD20). Moreover in immune deficient mice, bearing human B cell lymphoma xenografts, the combination of IL-21 + rituximab significantly increased survival, relative to either agent alone [122].

Different cytokines were shown to cooperate with IL-21 for antitumor activity. In nude mice, human ovarian cancer cells genetically modified to secrete both IL-21 and GM-CSF enhanced NK cell activity, IFN-*γ*, and tumor necrosis factor- (TNF-) *α* levels resulting in inhibition of tumor growth. The tumor inhibition effect was superior to that observed by the use of ovarian cancer cells expressing either cytokine alone [123]. In a syngeneic melanoma model a tumor cell vaccine that displayed membrane-anchored glycosylphosphatidylinositol- (GPI-) IL-21 delayed tumor growth and prolonged mice survival. Coexpression of GM-CSF further increased survival of melanoma-bearing mice by the activation of strong NK and CD8^+^ T cell responses [124]. Also, the addition of 6 kDa early secreted antigenic target (ESAT-6), a protein of* M. tuberculosis*, to B16/F10/GPI-IL-21 cell vaccine produced a powerful antimelanoma effect inhibiting tumor growth and prolonging mice survival. The efficacy of this cell vaccine was further increased by the administration of a DNA vaccine ESAT-6-GPI for priming, followed by B16/F10-ESAT-6-GPI/IL-21 to boost the immune response [125].

DNA vaccination is another interesting approach to induce an antigen-specific immune response towards tumor cells or pathogens, whose effects can be enhanced by IL-21. As an example, a DNA vaccine encoding for IL-21 and IL-15 genes linked to the 47-LDA mimotope of disialoganglioside GD2, an antigen expressed mainly in NB, was particularly effective in inhibiting tumor growth in mice bearing NXS2 GD2^+^ NB. The mechanism involved relied on the induction of an anti-GD2 IgG response as well as GD2-independent CD8^+^ T cell responses [126].

## 6. Use of IL-21 for Adoptive Cell Therapies

Adoptive immunotherapies require the in vitro expansion of lymphocytes endowed with antitumor properties, either spontaneous or induced by in vitro engineering, followed by reinfusion in the tumor-bearing host [127]. Whereas IL-2 or IL-15 has been widely utilized as growth factors for T cells, in conjunction with other cytokines, several data support important benefits in the usage of IL-21 in culture systems. An early report showed that IL-21 cooperates with IL-15 to promote the in vitro growth of both memory and naive mouse CD8^+^ T cells and in vivo expansion of antigen-specific CD8^+^ T cells mediating B16 melanoma regression [41]. Furthermore, IL-21 inhibited activation-induced eomesodermin expression in mouse CD8^+^ T cells and their maturation into differentiated effector cells. In addition, IL-21-cultured CD8^+^ T cells showed increased expression of L-selectin, enhanced antitumor functions, and long-term persistence upon adoptive transfer in syngeneic hosts. These effects of IL-21 stimulation in vitro were not reversed by subsequent activation with antigen and IL-2, thus supporting an important role for IL-21 in the development of CTL-based adoptive therapies for cancer [45]. Other reports showed that IL-21 combined with IL-15 preserved memory type features and CD28 expression of human CD8^+^ tumor-infiltrating lymphocytes (TILs) [128] or CTLs induced by antigen priming [39], allowing the generation of expanded CTL populations suitable for in vivo use. Human T cells genetically engineered to express a chimeric antigen receptor (CAR) specific for the CD19 B cell antigen and expanded in IL-21 containing medium displayed cytotoxicity and produced IFN-*γ* in response to CD19 antigen. These IL-21-expanded CD19-CAR^+^ T cells revealed an early memory phenotype (CD62L^+^CD28^+^) and effectively controlled human pre-B cell leukemia growth in immune deficient mice [129]. A similar method for the expansion of central memory-like (Tcm) TILs or T cells engineered to express a tumor-specific TCR is based on the use of IL-12 plus IL-7 or IL-21 followed by withdrawal of IL-12. This protocol resulted in the generation of T cells with a CD62L^high^CD28^high^CD127^high^CD27^high^CCR7^high^ phenotype, which expressed stem cell-related genes and persisted when injected in NOD/SCID/*γ*c^−/−^ mice. This culture protocol may therefore be highly suitable for adoptive immunotherapy of tumors [130]. A recent clinical study addressed the usage of rIL-21 in the culture system of WT-1-specific donor-derived CTLs that, after in vitro expansion, were reinfused in acute myeloid leukemia patients during the posttransplant period. Transfer of IL-21-treated CTL clones in four patients resulted in antileukemic activity in two of them: a patient with progressive disease showed a transient response while a patient with minimal residual disease had a prolonged remission. In addition, three other patients at high risk for relapse that were treated with IL-21-derived CTLs showed no leukemia relapse in the absence of additional treatments. CTLs generated in the presence of IL-21 were detectable at long-term and showed characteristics of long-lived memory CD8^+^ T cells in these patients [131].

The possible usage of IL-21 as a stimulus to sustain in vitro culture of human *γ*/*δ* T cells or NK cells has been also exploited. In an in vitro study, IL-21, in the presence of IL-2, enhanced the expansion of phosphoantigen-activated human V*γ*9V*δ*2 T cells and boosted their Th1-like program and cytotoxic activity against tumor cells [132]. In addition, IL-21, added to PBMC, which were stimulated with K562 cells expressing 4-1BB-L and MICA, increased the expansion of NKG2D^+^ NK cells. These NK cell populations produced IFN-*γ* and displayed enhanced antitumor cytotoxic activity [133]. Genetically engineered K562 cells expressing membrane-bound IL-21 support human NK cell proliferation at long-term without evidence of senescence, possibly due to an increase in telomere length. These results were superior to those obtained by the use of K562 cells expressing membrane IL-15. Although NK cells expanded with either membrane cytokine had similar cytotoxicity killer Ig-like receptors (KIR), natural cytotoxicity receptors (NCRs), CD16, and NKG2D expression, IL-21-expanded cells showed superior cytokine secretion and increased CD160 expression. K562-IL-21-expanded NK cells efficiently lysed tumor cells and displayed ADCC, supporting their potential use for NK adoptive immunotherapy [134]. In another report, K562 cells expressing membrane-bound IL-21, in association with CD137, demonstrated superior stimulation of NK cell cytotoxic activity in vitro [135]. In conclusion, IL-21, in combination with other cytokines (e.g., IL-15 or IL-2) or other stimulatory molecules, can support the in vitro expansion of different lymphoid cell populations endowed with enhanced antitumor properties, which are currently studied in clinical trials (e.g., NCT01787474, Table 1).

## 7. Clinical Studies of IL-21 in Cancer

In view of the efficacy of IL-21 in preclinical studies of tumor immunotherapy, clinical trials of IL-21 as a single agent or in combination with other drugs were performed, in different cancers (Table 1).

A phase I/IIa study of intravenous (iv) rIL-21, conducted in metastatic melanoma [136, 137], showed that IL-21 does not induce vascular-leak syndrome by repeated iv infusion. The maximal tolerated dose (MTD) for daily iv infusions was 30 *μ*g/kg, and dose-limiting toxicities consisted of hepatotoxicity, neutropenia, and lightheadedness with fever and rigors. IL-21 induced an increase in biomarkers of immune activation (soluble CD25, perforin, and granzyme B expression in CD8^+^ T and NK cells) at all dose levels. One complete and one partial response were also observed, suggesting clinical activity. Another phase I study on metastatic melanoma and renal cancer reported similar toxicities and the same MTD as the first study. In addition, the study reported one complete response and 11 disease stabilization in a melanoma cohort (24 patients) and four partial responses and 13 stabilizations in the renal cancer cohort [138]. A subsequent study of multianalytes was performed on sera of patients included in the two phase I studies. This study showed that IL-21 treatment increased IL-6, acute phase proteins, chemokines including macrophage-derived chemokine (MDC), macrophage inflammatory protein (MIP) 1a and monocyte chemoattractant protein- (MCP-) 1, and soluble cell adhesion molecules (CAMs). These data suggested the activation of immune/inflammatory responses by therapeutic IL-21 [139]. Another phase I study tested subcutaneous IL-21 at three doses per week for 8 or 16 weeks, in melanoma and renal cancer patients. The MTD was 200 *μ*g/kg and the toxicities were similar to those found in the other phase I studies. Subcutaneous IL-21 also increased biomarkers of immune activation and one melanoma and two renal cancer patients obtained partial responses (out of 26 total patients) [140]. A phase II trial of iv IL-21 was then conducted in 40 patients with metastatic melanoma. Nine out of 37 evaluable patients had partial responses (22.5%) and 16 had disease stabilizations, and the median overall survival was 12.4 months [141]. These data suggested that IL-21 had activity in metastatic melanoma and led to the design of a randomized phase II trial. This study enrolled 64 patients that were randomized to intravenous IL-21 or to dacarbazine (DTIC) chemotherapy. A preliminary report showed that, in spite of earlier encouraging results, IL-21 activity in metastatic melanoma is comparable to that of DTIC [142].

The acceptable toxicity and the low clinical activity suggested that IL-21 is suitable for combinational treatments with other agents. Based on preclinical studies, which indicated cooperative antitumor activity of IL-21 with mAbs targeting CD20 or epidermal growth factor receptor (EGF-R), two clinical studies of IL-21 combined with therapeutic mAbs were designed. A phase I study of weekly bolus tested IL-21 in combination with rituximab in patients with low-grade, relapsing B cell malignancies. Twenty-one patients with relapsed CLL, small lymphocytic, follicular, or marginal-zone lymphoma were enrolled. The MTD for IL-21 was 100 *μ*g/mL, and eight out of nineteen evaluable patients showed responses. The authors concluded that this treatment is safe and clinically active and may deserve further investigation [143]. A second phase I study tested IL-21 combined with cetuximab in stage IV colorectal cancer patients. The early closure of the study did not allow establishing an MTD. Nonetheless, this combination was safe, biomarkers of immune activation increased, and 60% of 16 treated patients had disease stabilization [144]. Another phase I study explored the combination of subcutaneous IL-21 with sunitinib, a kinase inhibitor, which induces partial responses in about one half of patients with metastatic renal cancer. However, the study was terminated due to hematologic dose-limiting toxicities (neutropenia and thrombocytopenia), encountered at the 10 *μ*g/kg IL-21 dose level, which was considered too low for expecting therapeutic effects [145]. Differently, a phase I/II trial reported that the combination of iv IL-21 with sorafenib, a multityrosine kinase inhibitor, is well-tolerated, in patients with metastatic renal carcinoma unresponsive to previous treatments. The IL-21 MTD was 30 *μ*g/kg with the standard dose of sorafenib, with skin rash as the dose-limiting toxicity. Soluble CD25 increased during treatment, indicating that sorafenib did not interfere with the immune-enhancing activity of IL-21. The objective response rate was 21%; disease control was achieved in 82% of patients and two persistent responses (41 and 30 months) continued after therapy withdrawal [146]. Therefore, IL-21 plus sorafenib has antitumor activity in previously treated metastatic renal cancer, and IL-21 should be further explored in combination therapies.

## 8. Concluding Remarks and Perspectives

Recent studies have demonstrated that IL-21, as many other interleukins, has a dual role in immune regulation. On one hand IL-21 has immune-enhancing properties, as it costimulates NK and CTL functions and is essential for driving IgG antibody production, as demonstrated by studies in* Il21* or* Il21r* ^−/−^ mice [17] and by the phenotype of immune deficient patients with analogous genetic defects [18, 19]. On the other hand, IL-21 elicits immune-regulatory circuits through the inhibition of DC functions [62], induction of IL-10 and Breg cells, which may limit the immune response [64, 69–71]. Nonetheless, IL-21 has been implicated in different immune-mediated diseases (e.g., SLE and inflammatory bowel diseases) due to its ability to drive the production of pathogenic autoantibodies [20–22, 28, 29, 33–36]. In addition, autocrine or paracrine effects of IL-21 have been described in different lymphoproliferative disorders [92–99]. In these diseases, blockade of the IL-21/IL-21R axis may represent a potential area of therapeutic intervention. To this end, a human anti-IL-21R blocking mAb, ATR-107, has been developed. However, a phase I study revealed a high immunogenic potential of this antibody, which seems to preclude further clinical applications [77]. Therefore, other blocking agents may be required to approach IL-21 functional blockade. A note of caution in using IL-21-blocking agents is related to the dual role of IL-21 in immune regulation, which may reflect opposite effects of IL-21 at various stages of disease development, as recently reported in a murine model of SLE [78].

IL-21 may also be exploited as therapeutic molecule to enhance immune functions in some infectious diseases and in some cancers. Indeed, IL-21 has shown to elicit antitumor immune responses in several preclinical tumor models [40, 100–116, 147]. This antitumor activity can be enhanced by combining IL-21 with other agents, which targets tumor cells (e.g., mAbs directed to tumor antigens) [120–122], immune-regulatory circuits (e.g., Treg cell blockade, immune-checkpoint blockers) [72, 115–119] or other immune-enhancing molecules (e.g., cytokines, immune-enhancing mAbs) [123–125].

Importantly, IL-21 represents a useful reagent, in combination with other cytokines, for in vitro expansion of CTLs or NK cells to be reinfused in tumor-bearing patients for adoptive cell therapy of cancer [38, 39, 51, 127–134]. In fact, IL-21 has the unique ability to induce a peculiar (CD28^+^CD127^high^) CTL memory phenotype in vitro, which results in long persistence of CTLs with antitumor activity upon in vivo reinfusion into leukemic patients, as shown in a recent study [131].

Clinical phase I/II studies have shown that IL-21 has acceptable toxicities and antitumor activity, leading to objective responses or disease stabilizations in a fraction of metastatic melanoma and renal cancer patients [136–141]. However, the response rate was similar to that of dacarbazine in a recent randomized clinical study [142]. Therefore, in view of its low activity and good tolerability, IL-21 is most likely suitable for combinational regimes of cancer immunotherapy. In fact, two clinical studies are addressing the possible cooperative effects of IL-21 with immune checkpoint blockers. A phase I study of IL-21 combined with the anti-CTLA4 mAb Ipilimumab in unresectable stage III or stage IV melanoma was recently completed (NCT01489059). The purpose of this study was to determine the safety of this combination and achieve preliminary information on clinical benefits compared with ipilimumab alone, but the results are not yet available. Finally a safety study of IL-21 with the anti-PD-1 mAb Nivolumab in advanced or metastatic solid tumors is ongoing (NCT01629758). It is hoped that these and similar studies will show clinical benefit of IL-21 in combinational therapies in cancer.

## Figures and Tables

**Table 1:** Clinical studies of IL-21 immunotherapy.

Clinical trial	Phase	Intervention	Status	Results
NN0281614	1	A two-arm phase I study of IL-21 in metastatic melanoma	Completed	MTD established at 30 *μ*g/kg. Increased biomarkers (sCD25, perforin granzyme). One PR that became CR 3 months later [136].

NCT00617253	1/2	Combination of rIL-21 and sunitinib in stage IV renal cell carcinoma patients	Completed	The tolerated dose of IL-21 (3 mg/kg) was too low and the study was discontinued [145].

NCT00095108	1/2	Study of IL-21 for metastatic malignant melanoma and metastatic kidney cancer	Completed	Melanoma: 1 CR and 11SD out of 24. Renal cancer: 4PR and 13SD out of 19 [138]

NCT00389285	1/2	Study of i.v. rIL-21 in combination with oral sorafenib for metastatic renal cell carcinoma	Completed	ORR 21% disease control rate 82% IL-21 plus sorafenib has antitumor activity and acceptable safety [146]

NCT00347971	1	Study of rIL-21 in combination with Rituxan for relapsed/refractory low-grade B cell lymphoma	Completed	ORR 42% (3 CR and 5PR out of 19 patients) [143]

NCT00514085	2	IL-21 in treating patients with metastatic or recurrent malignant melanoma	Completed	ORR 22.5% and 16 SD out of 40 patients [141]

NCT00336986	2	Efficacy study of IL-21 to treat metastatic melanoma	Completed	ORR 8%, increased biomarkers [137]

NCT00523380	2	Efficacy study of rIL-21 plus doxil in the treatment of ovarian cancer	Completed	No data available

NCT00601861	2	Effect of rIL-21 on metastases in lymph nodes in melanoma skin cancer	Terminated	No data available

NCT01152788	2	A randomized phase II study of rIL-21 versus dacarbazine in patients with metastatic or recurrent melanoma	Active, not recruiting	rIL-21 is comparable to DTIC in this patient population (4/30 = 13.3% 4/28 = 14.3%) [142]

NCT01489059	1	Safety study of IL-21/ipilimumab combination in the treatment of melanoma	Completed	No data available

NCT01629758	1	Safety study of IL-21/anti-PD-1 combination in the treatment of solid tumors	Active, not recruiting	No data available

NCT01787474	1/2	IL-21-expanded NK cells for induction of acute myeloid leukemia (AML)	Recruiting	No data available

## References

[1] Ozaki K., Kikly K., Michalovich D., Young P. R., Leonard W. J. (2000). Cloning of a type I cytokine receptor most related to the IL-2 receptor *β* chain. *Proceedings of the National Academy of Sciences of the United States of America*.

[2] Parrish-Novak J., Dillon S. R., Nelson A. (2000). Interleukin 21 and its receptor are involved in NK cell expansion and regulation of lymphocyte function. *Nature*.

[3] Rochman Y., Spolski R., Leonard W. J. (2009). New insights into the regulation of T cells by gammac family cytokines. *Nature Reviews Immunology*.

[4] Meazza R., Azzarone B., Orengo A. M., Ferrini S. (2011). Role of common-gamma chain cytokines in NK cell development and function: perspectives for immunotherapy. *Journal of Biomedicine and Biotechnology*.

[5] Spolski R., Leonard W. J. (2014). Interleukin-21: a double-edged sword with therapeutic potential. *Nature Reviews Drug Discovery*.

[6] Ghoreschi K., Laurence A., O'Shea J. J. (2009). Janus kinases in immune cell signaling. *Immunological Reviews*.

[7] Zeng R., Spolski R., Casas E., Zhu W., Levy D. E., Leonard W. J. (2007). The molecular basis of IL-21-mediated proliferation. *Blood*.

[8] de Totero D., Meazza R., Capaia M. (2008). The opposite effects of IL-15 and IL-21 on CLL B cells correlate with differential activation of the JAK/STAT and ERK1/2 pathways. *Blood*.

[9] Kwon H., Thierry-Mieg D., Thierry-Mieg J. (2009). Analysis of interleukin-21-induced Prdm1 gene regulation reveals functional cooperation of STAT3 and IRF4 transcription factors. *Immunity*.

[10] Jin H., Carrio R., Yu A., Malek T. R. (2004). Distinct activation signals determine whether IL-21 induces B cell costimulation, growth arrest, or Bim-dependent apoptosis. *Journal of Immunology*.

[11] Diehl S. A., Schmidlin H., Nagasawa M. (2008). STAT3-mediated Up-regulation of BLIMP1 is coordinated with BCL6 down-regulation to control human plasma cell differentiation. *The Journal of Immunology*.

[12] Strengell M., Lehtonen A., Matikainen S., Julkunen I. (2006). IL-21 enhances SOCS gene expression and inhibits LPS-induced cytokine production in human monocyte-derived dendritic cells. *Journal of Leukocyte Biology*.

[13] Jahrsdörfer B., Blackwell S. E., Wooldridge J. E. (2006). B-chronic lymphocytic leukemia cells and other B cells can produce granzyme B and gain cytotoxic potential after interleukin-21-based activation. *Blood*.

[14] Good K. L., Bryant V. L., Tangye S. G. (2006). Kinetics of human B cell behavior and amplification of proliferative responses following stimulation with IL-21. *Journal of Immunology*.

[15] Pène J., Gauchat J.-F., Lécart S. (2004). Cutting edge: IL-21 is a switch factor for the production of IgG_1_ and IgG_3_ by human B cells. *The Journal of Immunology*.

[16] Moens L., Tangye S. G. (2014). Cytokine-mediated regulation of plasma cell generation: IL-21 takes center stage. *Frontiers in Immunology*.

[17] Ozaki K., Spolski R., Feng C. G. (2002). A critical role for IL-21 in regulating immunoglobulin production. *Science*.

[18] Kotlarz D., Ziȩtara N., Uzel G. (2013). Loss-of-function mutations in the IL-21 receptor gene cause a primary immunodeficiency syndrome. *Journal of Experimental Medicine*.

[19] Salzer E., Kansu A., Sic H. (2014). Early-onset inflammatory bowel disease and common variable immunodeficiency-like disease caused by IL-21 deficiency. *Journal of Allergy and Clinical Immunology*.

[20] Ozaki K., Spolski R., Ettinger R. (2004). Regulation of B cell differentiation and plasma cell generation by IL-21, a novel inducer of Blimp-1 and Bcl-6. *Journal of Immunology*.

[21] Herber D., Brown T. P., Liang S., Young D. A., Collins M., Dunussi-Joannopoulos K. (2007). IL-21 has a pathogenic role in a lupus-prone mouse model and its blockade with IL-21R.Fc reduces disease progression. *The Journal of Immunology*.

[22] Sawalha A. H., Kaufman K. M., Kelly J. A. (2008). Genetic association of interleukin-21 polymorphisms with systemic lupus erythematosus. *Annals of the Rheumatic Diseases*.

[23] Nakano H., Kishida T., Asada H. (2006). Interleukin-21 triggers both cellular and humoral immune responses leading to therapeutic antitumor effects against head and neck squamous cell carcinoma. *The Journal of Gene Medicine*.

[24] Bauquet A. T., Jin H., Paterson A. M. (2009). The costimulatory molecule ICOS regulates the expression of c-Maf and IL-21 in the development of follicular T helper cells and TH -17 cells. *Nature Immunology*.

[25] Nurieva R. I., Chung Y., Martinez G. J. (2009). Bcl6 mediates the development of T follicular helper cells. *Science*.

[26] Bollig N., Brusütle A., Kellner K. (2012). Transcription factor IRF4 determines germinal center formation through follicular T-helper cell differentiation. *Proceedings of the National Academy of Sciences of the United States of America*.

[27] Sage P. T., Alvarez D., Godec J., von Andrian U. H., Sharpe A. H. (2014). Circulating T follicular regulatory and helper cells have memory-like properties. *Journal of Clinical Investigation*.

[28] Linterman M. A., Rigby R. J., Wong R. K. (2009). Follicular helper T cells are required for systemic autoimmunity. *The Journal of Experimental Medicine*.

[29] Odegard J. M., Marks B. R., Diplacido L. D. (2008). ICOS-dependent extrafollicular helper T cells elicit IgG production via IL-21 in systemic autoimmunity. *The Journal of Experimental Medicine*.

[30] Wei L., Laurence A., Elias K. M., O'Shea J. J. (2007). IL-21 is produced by Th17 cells and drives IL-17 production in a STAT3-dependent manner. *Journal of Biological Chemistry*.

[31] Nurieva R., Yang X. O., Martinez G. (2007). Essential autocrine regulation by IL-21 in the generation of inflammatory T cells. *Nature*.

[32] Zhou L., Ivanov I. I., Spolski R. (2007). IL-6 programs TH-17 cell differentiation by promoting sequential engagement of the IL-21 and IL-23 pathways. *Nature Immunology*.

[33] di Fusco D., Izzo R., Figliuzzi M. M., Pallone F., Monteleone G. (2014). IL-21 as a therapeutic target in inflammatory disorders. *Expert Opinion on Therapeutic Targets*.

[34] Monteleone G., Monteleone I., Fina D. (2005). Interleukin-21 enhances T-helper cell type I signaling and interferon-*γ* production in Crohn's disease. *Gastroenterology*.

[35] Sarra M., Monteleone I., Stolfi C. (2010). Interferon-gamma-expressing cells are a major source of interleukin-21 in inflammatory bowel diseases. *Inflammatory Bowel Diseases*.

[36] Fina D., Sarra M., Fantini M. C. (2008). Regulation of gut inflammation and Th17 cell response by interleukin-21. *Gastroenterology*.

[37] Li Y., Bleakley M., Yee C. (2005). IL-21 influences the frequency, phenotype, and affinity of the antigen-specific CD8 T cell response. *Journal of Immunology*.

[38] Alves N. L., Arosa F. A., van Lier R. A. W. (2005). IL-21 sustains CD28 expression on IL-15-activated human naive CD8^+^ T cells. *The Journal of Immunology*.

[39] Zeng R., Spolski R., Finkelstein S. E. (2005). Synergy of IL-21 and IL-15 in regulating CD8^+^ T cell expansion and function. *Journal of Experimental Medicine*.

[40] Di Carlo E., de Totero D., Piazza T., Fabbi M., Ferrini S. (2007). Role of IL-21 in immune-regulation and tumor immunotherapy. *Cancer Immunology, Immunotherapy*.

[41] Sutherland A. P. R., Joller N., Michaud M., Liu S. M., Kuchroo V. K., Grusby M. J. (2013). IL-21 promotes CD8^+^ CTL activity via the transcription factor T-bet. *The Journal of Immunology*.

[42] Gagnon J., Ramanathan S., Leblanc C., Ilangumaran S. (2007). Regulation of IL-21 signaling by suppressor of cytokine signaling-1 (SOCS1) in CD8^+^ T lymphocytes. *Cellular Signalling*.

[43] Hinrichs C. S., Spolski R., Paulos C. M. (2008). IL-2 and IL-21 confer opposing differentiation programs to CD8^+^ T cells for adoptive immunotherapy. *Blood*.

[44] van Leeuwen E. M. M., van Buul J. D., Remmerswaal E. B. M., Hordijk P. L., ten Berge I. J. M., van Lier R. A. W. (2005). Functional re-expression of CCR7 on CMV-specific CD8^+^ T cells upon antigenic stimulation. *International Immunology*.

[45] Fröhlich A., Kisielow J., Schmitz I. (2009). IL-21R on T cells is critical for sustained functionality and control of chronic viral infection. *Science*.

[46] Elsaesser H., Sauer K., Brooks D. G. (2009). IL-21 is required to control chronic viral infection. *Science*.

[47] Yi J. S., Du M., Zajac A. J. (2009). A vital role for Interleukin-21 in the control of a chronic viral infection. *Science*.

[48] Iannello A., Tremblay C., Routy J.-P., Boulassel M.-R., Toma E., Ahmad A. (2008). Decreased levels of circulating IL-21 in HIV-infected AIDS patients: correlation with CD4^+^ T-cell counts. *Viral Immunology*.

[49] Iannello A., Boulassel M.-R., Samarani S. (2010). Dynamics and consequences of IL-21 production in HIV-infected individuals: a longitudinal and cross-sectional study. *The Journal of Immunology*.

[50] Chevalier M. F., Jülg B., Pyo A. (2011). HIV-1-specific interleukin-21^+^ CD4^+^ T cell responses contribute to durable viral control through the modulation of HIV-specific CD8^+^ T cell function. *Journal of Virology*.

[51] White L., Krishnan S., Strbo N. (2007). Differential effects of IL-21 and IL-15 on perforin expression, lysosomal degranulation, and proliferation in CD8 T cells of patients with human immunodeficiency virus-1 (HIV). *Blood*.

[52] Williams L. D., Bansal A., Sabbaj S. (2011). Interleukin-21-producing HIV-1-specific CD8 T cells are preferentially seen in elite controllers. *Journal of Virology*.

[53] Williams L. D., Amatya N., Bansal A. (2014). Immune activation is associated with CD8 T cell interleukin-21 production in HIV-1-infected individuals. *Journal of Virology*.

[54] Toomey J. A., Gays F., Foster D., Brooks C. G. (2003). Cytokine requirements for the growth and development of mouse NK cells in vitro. *Journal of Leukocyte Biology*.

[55] Brady J., Hayakawa Y., Smyth M. J., Nutt S. L. (2004). IL-21 induces the functional maturation of murine NK cells. *The Journal of Immunology*.

[56] Kasaian M. T., Whitters M. J., Carter L. L. (2002). IL-21 limits NK cell responses and promotes antigen-specific T cell activation: a mediator of the transition from innate to adaptive immunity. *Immunity*.

[57] Zhu S., Phatarpekar P. V., Denman C. J. (2014). Transcription of the activating receptor NKG2D in natural killer cells is regulated by STAT3 tyrosine phosphorylation. *Blood*.

[58] Sivori S., Cantoni C., Parolini S. (2003). IL-21 induces both rapid maturation of human CD34+ cell precursors towards NK cells and acquisition of surface killer Ig-like receptors. *European Journal of Immunology*.

[59] Vosshenrich C. A. J., Ranson T., Samson S. I. (2005). Roles for common cytokine receptor *γ*-chain-dependent cytokines in the generation, differentiation, and maturation of NK cell precursors and peripheral NK cells in vivo. *The Journal of Immunology*.

[60] Watanabe M., Kono K., Kawaguchi Y. (2010). Interleukin-21 can efficiently restore impaired antibody-dependent cell-mediated cytotoxicity in patients with oesophageal squamous cell carcinoma. *British Journal of Cancer*.

[61] Roda J. M., Joshi T., Butchar J. P. (2007). The activation of natural killer cell effector functions by cetuximab-coated, epidermal growth factor receptor—positive tumor cells is enhanced by cytokines. *Clinical Cancer Research*.

[62] Brandt K., Bulfone-Paus S., Jenckel A., Foster D. C., Paus R., Rückert R. (2003). Interleukin-21 inhibits dendritic cell-mediated T cell activation and induction of contact hypersensitivity in vivo. *Journal of Investigative Dermatology*.

[63] Wan C.-K., Oh J., Li P. (2013). The cytokines IL-21 and GM-CSF have opposing regulatory roles in the apoptosis of conventional dendritic cells. *Immunity*.

[64] Spolski R., Kim H.-P., Zhu W., Levy D. E., Leonard W. J. (2009). IL-21 mediates suppressive effects via its induction of IL-10. *The Journal of Immunology*.

[65] Moore K. W., De Waal Malefyt R., Coffman R. L., O'Garra A. (2001). Interleukin-10 and the interleukin-10 receptor. *Annual Review of Immunology*.

[66] Jin J. O., Han X., Yu Q. (2013). Interleukin-6 induces the generation of IL-10-producing Tr1 cells and suppresses autoimmune tissue inflammation. *Journal of Autoimmunity*.

[67] Doganci A., Birkholz J., Gehring S., Puhl A. G., Zepp F., Meyer C. U. (2013). In the presence of IL-21 human cord blood T cells differentiate to IL-10-producing Th1 but not Th17 or Th2 cells. *International Immunology*.

[68] Pot C., Jin H., Awasthi A. (2009). Cutting edge: IL-27 induces the transcription factor c-Maf, cytokine IL-21, and the costimulatory receptor ICOS that coordinately act together to promote differentiation of IL-10-producing Tr1 cells. *The Journal of Immunology*.

[69] He Y., Qian H., Liu Y., Duan L., Li Y., Shi G. (2014). The roles of regulatory B cells in cancer. *Journal of Immunology Research*.

[70] Yoshizaki A., Miyagaki T., Dilillo D. J. (2012). Regulatory B cells control T-cell autoimmunity through IL-21-dependent cognate interactions. *Nature*.

[71] Lindner S., Dahlke K., Sontheimer K. (2013). Interleukin 21-induced granzyme B-expressing B cells infiltrate tumors and regulate T cells. *Cancer Research*.

[72] Comes A., Rosso O., Orengo A. M. (2006). CD25^+^ regulatory T cell depletion augments immunotherapy of micrometastases by an IL-21-secreting cellular vaccine. *Journal of Immunology*.

[73] Peluso I., Fantini M. C., Fina D. (2007). IL-21 counteracts the regulatory T cell-mediated suppression of human CD4^+^ T lymphocytes. *Journal of Immunology*.

[74] Attridge K., Wang C. J., Wardzinski L. (2012). IL-21 inhibits T cell IL-2 production and impairs Treg homeostasis. *Blood*.

[75] Battaglia A., Buzzonetti A., Baranello C. (2013). Interleukin-21 (IL-21) synergizes with IL-2 to enhance T-cell receptor-induced human T-cell proliferation and counteracts IL-2/transforming growth factor-*β*-induced regulatory T-cell development. *Immunology*.

[76] Terrier B., Geri G., Chaara W. (2012). Interleukin-21 modulates Th1 and Th17 responses in giant cell arteritis. *Arthritis & Rheumatism*.

[77] Hua F., Comer G. M., Stockert L. (2014). Anti-IL21 receptor monoclonal antibody (ATR-107): safety, pharmacokinetics, and pharmacodynamic evaluation in healthy volunteers: a phase 1, first-in-human study. *The Journal of Clinical Pharmacology*.

[78] McPhee C. G., Bubier J. A., Sproule T. J. (2013). IL-21 is a double-edged sword in the systemic lupus erythematosus-like disease of BXSB.Yaa mice. *The Journal of Immunology*.

[79] Ma J., Ma D., Ji C. (2011). The role of IL-21 in hematological malignancies. *Cytokine*.

[80] Zhang S., Kipps T. J. (2014). The pathogenesis of chronic lymphocytic leukemia. *Annual Review of Pathology: Mechanisms of Disease*.

[81] Burger J. A., Gribben J. G. (2014). The microenvironment in chronic lymphocytic leukemia (CLL) and other B cell malignancies: insight into disease biology and new targeted therapies. *Seminars in Cancer Biology*.

[82] De Totero D., Meazza R., Zupo S. (2006). Interleukin-21 receptor (IL-21R) is up-regulated by CD40 triggering and mediates proapoptotic signals in chronic lymphocytic leukemia B cells. *Blood*.

[83] Gowda A., Roda J., Hussain S.-R. A. (2008). IL-21 mediates apoptosis through up-regulation of the BH3 family member BIM and enhances both direct and antibody-dependent cellular cytotoxicity in primary chronic lymphocytic leukemia cells in vitro. *Blood*.

[84] Hagn M., Blackwell S. E., Beyer T. (2014). B-CLL cells acquire APC- and CTL-like phenotypic characteristics after stimulation with CpG ODN and IL-21. *International Immunology*.

[85] Pascutti M. F., Jak M., Tromp J. M. (2013). IL-21 and CD40L signals from autologous T cells can induce antigen-independent proliferation of CLL cells. *Blood*.

[86] Ahearne M. J., Willimott S., Piñon L. (2013). Enhancement of CD154/IL4 proliferation by the T follicular helper (Tfh) cytokine, IL21 and increased numbers of circulating cells resembling Tfh cells in chronic lymphocytic leukaemia. *British Journal of Haematology*.

[87] Akamatsu N., Yamada Y., Hasegawa H. (2007). High IL-21 receptor expression and apoptosis induction by IL-21 in follicular lymphoma. *Cancer Letters*.

[88] de Totero D., Capaia M., Fabbi M. (2010). Heterogeneous expression and function of IL-21R and susceptibility to IL-21-mediated apoptosis in follicular lymphoma cells. *Experimental Hematology*.

[89] Wood B., Sikdar S., Choi S. J. (2013). Abundant expression of interleukin-21 receptor in follicular lymphoma cells is associated with more aggressive disease. *Leukemia & Lymphoma*.

[90] Sarosiek K. A., Malumbres R., Nechushtan H., Gentles A. J., Avisar E., Lossos I. S. (2010). Novel IL-21 signaling pathway up-regulates c-Myc and induces apoptosis of diffuse large B-cell lymphomas. *Blood*.

[91] Gelebart P., Zak Z., Anand M., Dien-Bard J., Amin H. M., Lai R. (2009). Interleukin-21 effectively induces apoptosis in mantle cell lymphoma through a STAT1-dependent mechanism. *Leukemia*.

[92] Bard J. D., Gelebart P., Anand M. (2009). IL-21 contributes to JAK3/STAT3 activation and promotes cell growth in ALK-positive anaplastic large cell lymphoma. *American Journal of Pathology*.

[93] Scheeren F. A., Diehl S. A., Smit L. A. (2008). IL-21 is expressed in Hodgkin lymphoma and activates STAT5: evidence that activated STAT5 is required for Hodgkin lymphomagenesis. *Blood*.

[94] Brenne A.-T., Ro T. B., Waage A., Sundan A., Borset M., Hjorth-Hansen H. (2002). Interleukin-21 is a growth and survival factor for human myeloma cells. *Blood*.

[95] Ménoret E., Maïga S., Descamps G. (2008). IL-21 stimulates human myeloma cell growth through an autocrine IGF-1 loop. *The Journal of Immunology*.

[96] Hodge L. S., Ziesmer S. C., Yang Z. Z. (2012). IL-21 in the bone marrow microenvironment contributes to IgM secretion and proliferation of malignant cells in Waldenstrom macroglobulinemia. *Blood*.

[97] Ueda M., Imada K., Imura A., Koga H., Hishizawa M., Uchiyama T. (2005). Expression of functional interleukin-21 receptor on adult T-cell leukaemia cells. *British Journal of Haematology*.

[98] Marzec M., Liu X., Wysocka M., Rook A. H., Odum N., Wasik M. A. (2011). Simultaneous inhibition of mTOR-containing complex 1 (mTORC1) and MNK induces apoptosis of cutaneous T-cell Lymphoma (CTCL) cells. *PLoS ONE*.

[99] van der Fits L., Out-Luiting J. J., Tensen C. P., Zoutman W. H., Vermeer M. H. (2014). Exploring the IL-21-STAT3 axis as therapeutic target for Sézary syndrome. *Journal of Investigative Dermatology*.

[100] di Carlo E., Comes A., Orengo A. M. (2004). IL-21 induces tumor rejection by specific CTL and IFN-gamma-dependent CXC chemokines in syngeneic mice. *The Journal of Immunology*.

[101] Ma H.-L., Whitters M. J., Konz R. F. (2003). IL-21 activates both innate and adaptive immunity to generate potent antitumor responses that require perforin but are independent of IFN-gamma. *The Journal of Immunology*.

[102] Ugai S.-I., Shimozato O., Kawamura K. (2003). Expression of the interleukin-21 gene in murine colon carcinoma cells generates systemic immunity in the inoculated hosts. *Cancer Gene Therapy*.

[103] Kumano M., Hara I., Furukawa J. (2007). Interleukin-21 activates cytotoxic T lymphocytes and natural killer cells to generate antitumor response in mouse renal cell carcinoma. *The Journal of Urology*.

[104] Furukawa J., Hara I., Nagai H., Yao A., Oniki S., Fujisawa M. (2006). Interleukin-21 gene transfection into mouse bladder cancer cells results in tumor rejection through the cytotoxic T lymphocyte response. *Journal of Urology*.

[105] Daga A., Orengo A. M., Gangemi R. M. R. (2007). Glioma immunotherapy by IL-21 gene-modified cells or by recombinant IL-21 involves antibody responses. *International Journal of Cancer*.

[106] Dou J., Wu Y., Wang J. (2010). Eliciting protective immune responses against murine myeloma challenge in lymphopenia mice through adoptive transfer of tumor antigen-specific lymphocytes and immunization of tumor vaccine secreting mIL-21. *Cancer Gene Therapy*.

[107] Aravindaram K., Wang P.-H., Yin S.-Y., Yang N.-S. (2014). Tumor-associated antigen/IL-21-transduced dendritic cell vaccines enhance immunity and inhibit immunosuppressive cells in metastatic melanoma. *Gene Therapy*.

[108] Hu W., Wang J., Dou J. (2011). Augmenting therapy of ovarian cancer efficacy by secreting IL-21 human umbilical cord blood stem cells in nude mice. *Cell Transplantation*.

[109] Moroz A., Eppolito C., Li Q., Tao J., Clegg C. H., Shrikant P. A. (2004). IL-21 enhances and sustains CD8^+^ T cell responses to achieve durable tumor immunity: comparative evaluation of IL-2, IL-15, and IL-21. *The Journal of Immunology*.

[110] Søndergaard H., Galsgaard E. D., Bartholomaeussen M., Straten P. T., Ødum N., Skak K. (2010). Intratumoral interleukin-21 increases antitumor immunity, tumor-infiltrating CD8^+^ T-cell density and activity, and enlarges draining lymph nodes. *Journal of Immunotherapy*.

[111] Sivakumar P. V., Garcia R., Waggie K. S., Anderson-Haley M., Nelson A., Hughes S. D. (2013). Comparison of vascular leak syndrome in mice treated with IL21 or IL2. *Comparative Medicine*.

[112] Skak K., Kragh M., Hausman D., Smyth M. J., Sivakumar P. V. (2008). Interleukin 21: combination strategies for cancer therapy. *Nature Reviews Drug Discovery*.

[113] Whiteside T. L. (2014). Clinical impact of regulatory T cells (Treg) in cancer and HIV. *Cancer Microenvironment*.

[114] Colombo M. P., Piconese S. (2007). Regulatory-T-cell inhibition versus depletion: the right choice in cancer immunotherapy. *Nature Reviews Cancer*.

[115] Croce M., Corrias M. V., Orengo A. M. (2010). Transient depletion of CD4^+^ T cells augments IL-21-based immunotherapy of disseminated neuroblastoma in syngeneic mice. *International Journal of Cancer*.

[116] Rigo V., Corrias M. V., Orengo A. M. (2014). Recombinant IL-21 and anti-CD4 antibodies cooperate in syngeneic neuroblastoma immunotherapy and mediate long-lasting immunity. *Cancer Immunology, Immunotherapy*.

[117] Pardoll D. M. (2012). The blockade of immune checkpoints in cancer immunotherapy. *Nature Reviews Cancer*.

[118] Pan X.-C., Li L., Mao J.-J. (2013). Synergistic effects of soluble PD-1 and IL-21 on antitumor immunity against H22 murine hepatocellular carcinoma. *Oncology Letters*.

[119] Jure-Kunkel M., Masters G., Girit E. (2013). Synergy between chemotherapeutic agents and CTLA-4 blockade in preclinical tumor models. *Cancer Immunology, Immunotherapy*.

[120] Smyth M. J., Teng M. W. L., Sharkey J. (2008). Interleukin 21 enhances antibody-mediated tumor rejection. *Cancer Research*.

[121] Roda J. M., Parihar R., Lehman A., Mani A., Tridandapani S., Carson W. E. (2006). Interleukin-21 enhances NK cell activation in response to antibody-coated targets. *The Journal of Immunology*.

[122] Krejsa C. M., Holly R. D., Heipel M. (2013). Interleukin-21 enhances rituximab activity in a cynomolgus monkey model of B cell depletion and in mouse B cell lymphoma models. *PLoS ONE*.

[123] Dou J., Wang Y., Wang J. (2009). Antitumor efficacy induced by human ovarian cancer cells secreting IL-21 alone or combination with GM-CSF cytokines in nude mice model. *Immunobiology*.

[124] Zhao F., Dou J., He X. F. (2010). Enhancing therapy of B16F10 melanoma efficacy through tumor vaccine expressing GPI-anchored IL-21 and secreting GM-CSF in mouse model. *Vaccine*.

[125] He X., Wang J., Zhao F. (2013). ESAT-6-gpi DNA vaccine augmented the specific antitumour efficacy induced by the tumour vaccine B16F10-ESAT-6-gpi/IL-21 in a mouse model. *Scandinavian Journal of Immunology*.

[126] Kowalczyk A., Wierzbicki A., Gil M. (2007). Induction of protective immune responses against NXS2 neuroblastoma challenge in mice by immunotherapy with GD2 mimotope vaccine and IL-15 and IL-21 gene delivery. *Cancer Immunology, Immunotherapy*.

[127] Wu R., Forget M.-A., Chacon J. (2012). Adoptive T-cell therapy using autologous tumor-infiltrating lymphocytes for metastatic melanoma: current status and future outlook. *The Cancer Journal*.

[128] Li Y., Liu S., Hernandez J., Vence L., Hwu P., Radvanyi L. (2010). MART-1-specific melanoma tumor-infiltrating lymphocytes maintaining CD28 expression have improved survival and expansion capability following antigenic restimulation in vitro. *The Journal of Immunology*.

[129] Singh H., Figliola M. J., Dawson M. J. (2011). Reprogramming CD19-specific T cells with IL-21 signaling can improve adoptive immunotherapy of B-lineage malignancies. *Cancer Research*.

[130] Yang S., Ji Y., Gattinoni L. (2013). Modulating the differentiation status of ex vivo-cultured anti-tumor T cells using cytokine cocktails. *Cancer Immunology, Immunotherapy*.

[131] Chapuis A. G., Ragnarsson G. B., Nguyen H. N. (2013). Transferred WT1-reactive CD8^+^ T cells can mediate antileukemic activity and persist in post-transplant patients. *Science Translational Medicine*.

[132] Thedrez A., Harly C., Morice A., Salot S., Bonneville M., Scotet E. (2009). IL-21-mediated potentiation of antitumor cytolytic and proinflammatory responses of human Vgamma9Vdelta2 T cells for adoptive immunotherapy. *The Journal of Immunology*.

[133] Jiang B., Wu X., Li X.-N. (2014). Expansion of NK cells by engineered K562 cells co-expressing 4-1BBL and mMICA, combined with soluble IL-21. *Cellular Immunology*.

[134] Denman C. J., Senyukov V. V., Somanchi S. S. (2012). Membrane-bound IL-21 promotes sustained ex vivo proliferation of human natural killer cells. *PLoS ONE*.

[135] Wang X., Lee D. A., Wang Y. (2013). Membrane-bound interleukin-21 and CD137 ligand induce functional human natural killer cells from peripheral blood mononuclear cells through STAT-3 activation. *Clinical & Experimental Immunology*.

[136] Davis I. D., Skrumsager B. K., Cebon J. (2007). An open-label, two-arm, phase I trial of recombinant human interleukin-21 in patients with metastatic melanoma. *Clinical Cancer Research*.

[137] Davis I. D., Brady B., Kefford R. F. (2009). Clinical and biological efficacy of recombinant human interleukin-21 in patients with stage IV Malignant melanoma without prior treatment: a phase IIa trial. *Clinical Cancer Research*.

[138] Thompson J. A., Curti B. D., Redman B. G. (2008). Phase I study of recombinant interleukin-21 in patients with metastatic melanoma and renal cell carcinoma. *Journal of Clinical Oncology*.

[139] Dodds M. G., Frederiksen K. S., Skak K. (2009). Immune activation in advanced cancer patients treated with recombinant IL-21: multianalyte profiling of serum proteins. *Cancer Immunology, Immunotherapy*.

[140] Schmidt H., Brown J., Mouritzen U. (2010). Safety and clinical effect of subcutaneous human interleukin-21 in patients with metastatic melanoma or renal cell carcinoma: a phase I trial. *Clinical Cancer Research*.

[141] Petrella T. M., Tozer R., Belanger K. (2012). Interleukin-21 has activity in patients with metastatic melanoma: a phase II study. *Journal of Clinical Oncology*.

[142] Petrella T. M., Mihalcioiu C. L. D., McWhirter E. (2013). Final efficacy results of NCIC CTG IND.202: a randomized phase II study of recombinant interleukin-21 (rIL21) in patients with recurrent or metastatic melanoma (MM). *Journal of Clinical Oncology*.

[143] Timmerman J. M., Byrd J. C., Andorsky D. J. (2012). A phase I dose-finding trial of recombinant interleukin-21 and rituximab in relapsed and refractory low grade B-cell lymphoproliferative disorders. *Clinical Cancer Research*.

[144] Steele N., Anthony A., Saunders M. (2012). A phase 1 trial of recombinant human IL-21 in combination with cetuximab in patients with metastatic colorectal cancer. *British Journal of Cancer*.

[145] Grünwald V., Desar I. M. E., Haanen J. (2011). A Phase i study of recombinant human interleukin-21 (rIL-21) in combination with sunitinib in patients with metastatic renal cell carcinoma (RCC). *Acta Oncologica*.

[146] Bhatia S., Curti B., Ernstoff M. S. (2014). Recombinant interleukin-21 plus sorafenib for metastatic renal cell carcinoma: a phase 1/2 study. *Journal for ImmunoTherapy of Cancer*.

[147] Croce M., Meazza R., Orengo A. M. (2008). Immunotherapy of neuroblastoma by an Interleukin-21-secreting cell vaccine involves survivin as antigen. *Cancer Immunology, Immunotherapy*.

